# Transcriptional E2F1/2/5/8 as potential targets and transcriptional E2F3/6/7 as new biomarkers for the prognosis of human lung carcinoma

**DOI:** 10.18632/aging.101441

**Published:** 2018-05-11

**Authors:** Cheng-Cao Sun, Qun Zhou, Wei Hu, Shu-Jun Li, Feng Zhang, Zhen-Long Chen, Guang Li, Zhuo-Yue Bi, Yong-Yi Bi, Feng-Yun Gong, Tao Bo, Zhan-Peng Yuan, Wei-Dong Hu, Bo-Tao Zhan, Qian Zhang, Qi-Zhu Tang, De-Jia Li

**Affiliations:** 1 Department of Preventive Medicine, School of Health Sciences, Wuhan University, Wuhan 430071, Hubei, P.R. China; 2 Department of Molecular and Cellular Oncology, The University of Texas MD Anderson Cancer Center, Houston, TX 77030, USA; 3 Department of Occupational and Environmental Health, School of Health Sciences, Wuhan University, Wuhan 430071, Hubei, P.R. China; 4 Wuhan Hospital for the Prevention and Treatment of Occupational Diseases, Wuhan 430022, Hubei, P.R. China; 5 Department of Oncology, Wuhan Pu-Ai Hospital, Tongji Medical College, Huazhong University of Science and Technology, Wuhan 430034, Hubei, P.R. China; 6 Hubei Provincial Key Laboratory for Applied Toxicology (Hubei Provincial Academy for Preventive Medicine), Wuhan 430079, Hubei, P.R. China; 7 Department of Infectious Diseases, Wuhan Medical Treatment Center, Wuhan 430023, Hubei, P.R. China; 8 Department of Oncology, ZhongNan Hospital of Wuhan University, Wuhan 430071, Hubei, P.R. China; 9 Xiangyang Central Hospital, Affiliated Hospital of Hubei University of Arts and Science, Xiangyang 430021, Hubei, P.R. China; 10 Department of Pathology, The Central Hospital of Wuhan, Tongji Medical College, Huazhong University of Science and Technology, Wuhan 430014, Hubei, P.R. China; 11 Department of Cardiology, Renmin Hospital of Wuhan University, Wuhan 430060, Hubei, P.R. China

**Keywords:** E2F translational factors, lung carcinoma, prognosis, Kaplan-Meier plot

## Abstract

E2F is a group of genes that encode a family of transcription factors (TFs) in higher eukaryotes and participate in cell cycle regulation and DNA synthesis in mammalian cells. Evidence from cell lines, mouse models, and human tissues indicates that TFs are implicated in lung cancer (LC) tumorigenesis. However, the diverse expression patterns and prognostic values of eight E2Fs have yet to be elucidated. In the current study, we examined the transcriptional and survival data of E2Fs in patients with LC from ONCOMINE, GEPIA, Kaplan–Meier Plotter, and cBioPortal databases. We found that the expression levels of E2F1/2/3/5/6/7/8 were higher in lung adenocarcinoma and squamous cell lung carcinoma tissues than in lung tissues, whereas the expression level of E2F4 was lower in the former than in the latter. The expression levels of E2F2/4/5/7/8 were correlated with advanced tumor stage. Survival analysis using the Kaplan–Meier Plotter database revealed that the high transcription levels of E2F1/2/4/5/7/8 were associated with low relapse-free survival (RFS) in all of the patients with LC. Conversely, high E2F3/6 levels predicted high RFS in these patients. This study implied that E2F3/6/7 are potential targets of precision therapy for patients with LC and that E2F1/2/4/5/8 are new biomarkers for the prognosis of LC.

## Introduction

E2Fs, a group of genes that encode a family of transcription factors (TFs) in higher eukaryotes, are generally subdivided into two groups based on functions: transcriptional activators (E2F1, E2F2, and E2F3a) and transcriptional repressors (E2F3b and E2F4-8) [1]. E2F family members play a major role in cell cycle regulation and DNA synthesis in mammalian cells [2]. The expression of E2F activators is deregulated in several human malignancies, including bladder cancer [3], breast cancer [4], ovarian cancer [5], prostate cancer [5], gastrointestinal cancer [6], and lung cancer [7,8].

Lung cancer (LC) is a common malignancy and the leading cause of cancer-related deaths worldwide [9–11]. This malignancy is divided into two main histological types: non-small cell lung cancer (NSCLC) and small cell lung cancer (SCLC). NSCLC includes adenocarcinoma, squamous cell carcinoma, and large cell carcinoma and accounts for approximately 85% of all LCs [12–15]. Despite considerable advancements in diagnostic and treatment methods, the 5-year overall survival rate of LC remains less than 15% [16,17]. Hence, prognostic markers and potential drug targets should be identified to enhance prognosis and individualized treatments.

Up to date, eight E2F factors have been identified in mammalian cells and numbered in the order of their discovery (E2F1, E2F2, E2F3, E2F4, E2F5, E2F6, E2F7, and E2F8) [4,6]. Among these factors, E2F1, E2F2, E2F3, and E2F8 are considered oncogenes in LC development, as indicated by evidence in LC cell lines, animal models, and primary human tissues [7,8,18–20]. Huang et al. reported that E2F1 gene expression is correlated with TS and Survivin gene expression and tumor proliferation. During the progression of NSCLC, E2F1 overexpression can result in increased aggressiveness, high proliferation rate, and enhanced chemoresistance in tumors [7]. Park et al. [19] showed that E2F8 is overexpressed in LC tumors compared with that in normal lung tissues, and the depletion of E2F8 inhibits LC cell proliferation and tumor growth by suppressing UHRF1 expression through UHRF1 promoter binding. However, the underlying mechanism by which E2Fs are activated or depressed and the distinct functions of the E2F factors in LC have yet to be fully elucidated.

The dysregulated expression level of E2F factors and their relationship with clinicopathological features and prognosis have been partly reported in human LC. To the best of our knowledge, bioinformatics analysis has yet been applied to explore the role of E2Fs in LC. RNA and DNA research, an essential component of biological and biomedical studies, have been revolutionized with the development of microarray technology [21]. On the basis of the analyses of thousands of gene expression or variation in copy numbers published online, we analyzed the expression and mutations of different E2F factors in patients with LC in detail to determine the expression patterns, potential functions, and distinct prognostic values of TFs in LC.

## RESULTS

### Transcriptional levels of E2Fs in patients with LC

**Figure 1:**
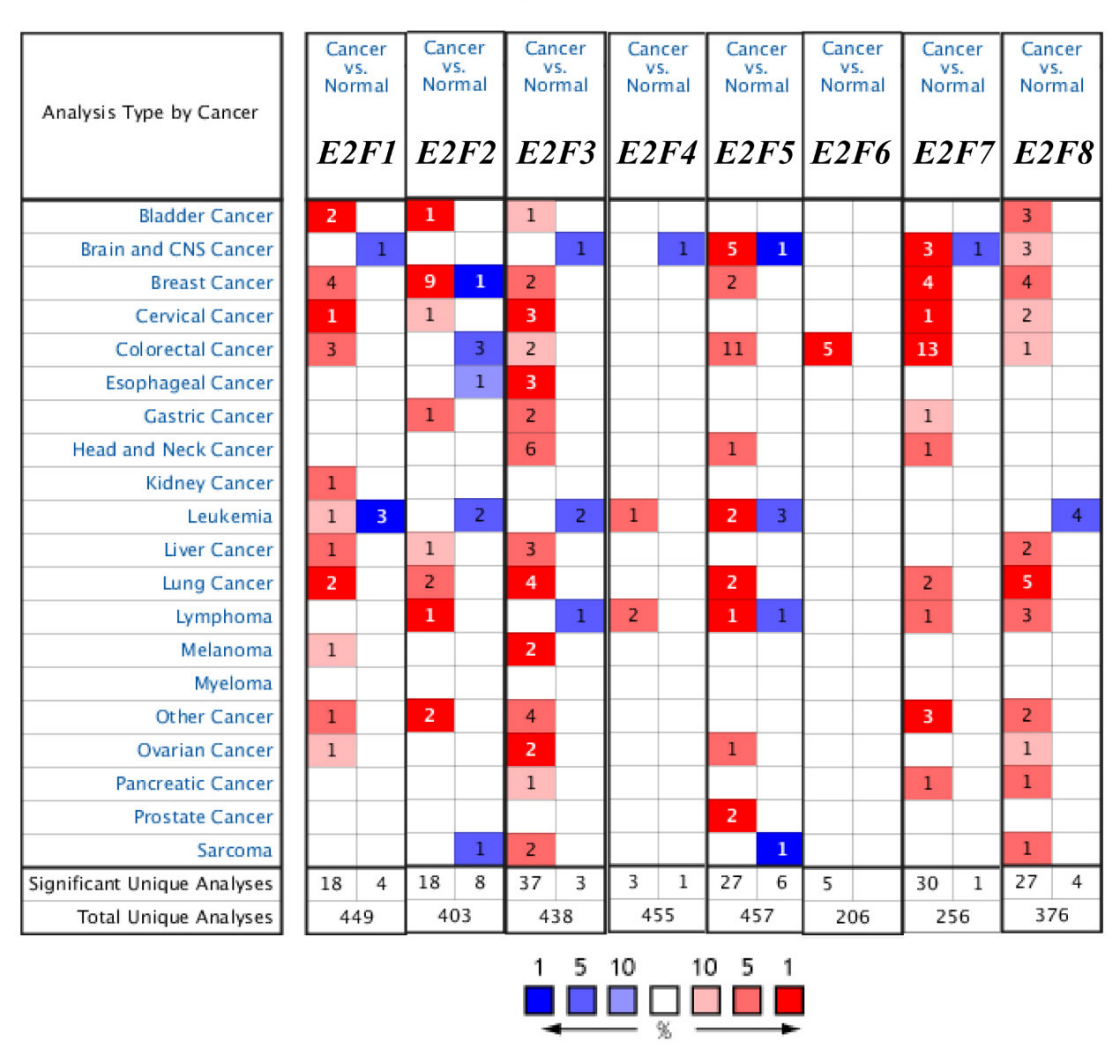
The transcription levels of E2F factors in different types of cancers (ONCOMINE).

Eight E2F factors have been identified in mammalian cells. We compared the transcriptional levels of E2Fs in cancers with those in normal samples by using ONCOMINE databases (Figure 1). The mRNA expression levels of E2F8 were significantly upregulated in patients with LC in five datasets. In Hou’s dataset [22], E2F8 is overexpressed compared with that in the normal samples in all of the LC types: lung adenocarcinoma with a fold change of 3.659, large-cell lung carcinoma with a fold change of 4.707, and squamous cell lung carcinoma with a fold change of 2.48 (Table 1). In Su’s dataset [23], E2F8 is also overexpressed in lung adenocarcinoma with a fold change of 5.779. Bhattacharjee [24] showed another mRNA expression factor with increased expression; that is, E2F3 has a fold change of 3.002 in patients with lung adenocarcinoma and a fold change of 3.002 in patients with SCLC compared with that in patients with normal lung tissues (Table 1). E2F3 overexpression is also found in large-cell lung carcinoma with a fold change of 2.338 in Hou’s dataset [22] and in SCLC with a fold change of 2.006 in Talbot’s dataset [25].

**Table 1:** The significant changes of E2Fs expression in transcription level between different types of lung cancer and lung tissues (ONCOMINE database)

	Types of lung cancer vs. lung	Fold change	P value	t-test	Ref
E2F1	Lung Adenocarcinoma vs. Normal	2.142	2.79E-06	6.168	Garber [26]
	Squamous Cell Lung Carcinoma vs. Normal	2.084	2.21E-05	5.658	Garber [26]
E2F2	Lung Adenocarcinoma vs. Normal	2.238	1.12E-14	9.707	Selamat [27]
E2F3	Squamous Cell Lung Carcinoma vs. Normal	2.066	1.66E-13	9.337	Talbot [25]
	Lung Adenocarcinoma vs. Normal	3.002	7.14E-05	4.733	Bhattacharjee [24]
	Small Cell Lung Carcinoma vs. Normal	4.391	7.16E-06	5.632	Bhattacharjee [24]
	Large Cell Lung Carcinoma vs. Normal	2.338	4.91E-06	5.904	Hou [22]
E2F4	NA	NA	NA	NA	NA
E2F5	Lung Adenocarcinoma vs. Normal	4.085	9.91E-05	4.576	Bhattacharjee [24]
	Lung Adenocarcinoma vs. Normal	2.208	1.51E-06	5.984	Stearman [28]
E2F6	NA	NA	NA	NA	NA
E2F7	Squamous Cell Lung Carcinoma vs. Normal	7.296	1.46E-13	12.969	Hou [22]
	Large Cell Lung Carcinoma vs. Normal	4.243	1.20E-05	5.597	Hou [22]
E2F8	Lung Adenocarcinoma vs. Normal	5.779	4.87E-11	8.272	Su [23]
	Lung Adenocarcinoma vs. Normal	3.659	1.51E-12	9.277	Hou [22]
	Large Cell Lung Carcinoma vs. Normal	4.707	2.43E-06	6.356	Hou [22]
	Squamous Cell Lung Carcinoma vs. Normal	2.48	4.96E-10	8.722	Hou [22]

The mRNA expression levels of E2F1, E2F2, E2F5, and E2F7 were upregulated in patients with LC. The transcription levels of E2F1 in lung adenocarcinoma and squamous cell lung carcinoma are higher than those in lung tissues, and their fold changes are 2.142 and 2.084, respectively [26]. In Selamat’s dataset [27], the mRNA expression of E2F2 in lung adenocarcinoma increases with a fold change of 2.238 (*p* < 0.005). A similar trend is showed in E2F5 in Bhattacharjee’s [24] and Stearman’s datasets [28]. E2F5 is significantly upregulated in lung adenocarcinoma, with fold changes of 4.085 and 2.208 in Bhattacharjee’s dataset [24] and Stearman’s dataset [28], respectively (Table 1). The transcriptional levels of E2F7 in squamous cell lung carcinoma (fold change = 7.296) and in large-cell lung carcinoma (with fold change = 4.243) significantly differ from those in the normal samples in Hou’s dataset (Table 1) [22].

### Relationship between the mRNA levels of E2Fs and the clinicopathological parameters of patients with LC

**Figure 2:**
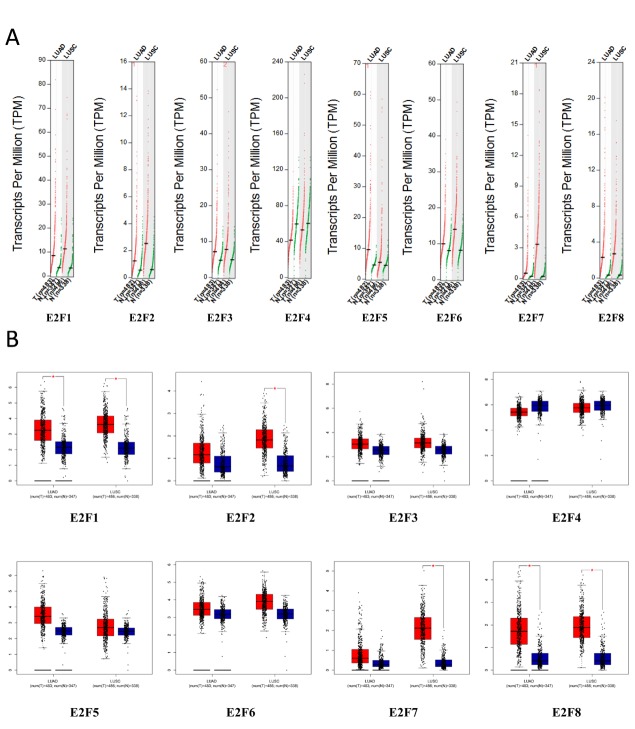
The expression of E2Fs in LC (GEPIA).

**Figure 3:**
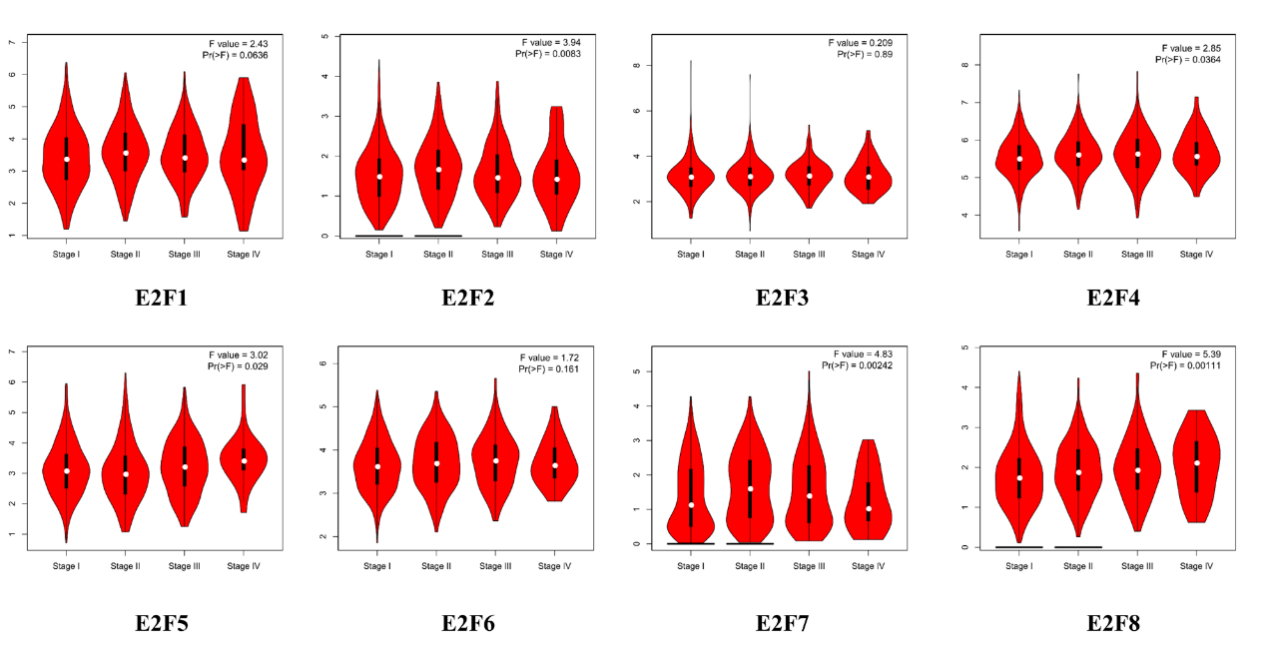
Correlation between E2Fs expression and tumor stage in LC patients (GEPIA).

Using GEPIA (Gene Expression Profiling Interactive Analysis) dataset (http://gepia.cancer-pku.cn/), we compared the mRNA expression of E2F factors between LC and lung tissues. The results indicated that the expression levels of E2F1, E2F2, E2F3, E2F5, E2F6, E2F7, and E2F8 were higher in lung adenocarcinoma and squamous cell lung carcinoma tissues than in lung tissues, whereas and the expression level of E2F4 was lower in the former than in the latter (Figure 2). We also analyzed the expression of E2Fs with tumor stage for lung adenocarcinoma and squamous cell lung carcinoma. E2F2, E2F4, E2F5, E2F7, and E2F8 groups significantly varied, whereas E2F1, E2F3, and E2F6 groups did not significantly differ (Figure 3).

**Figure 4:**
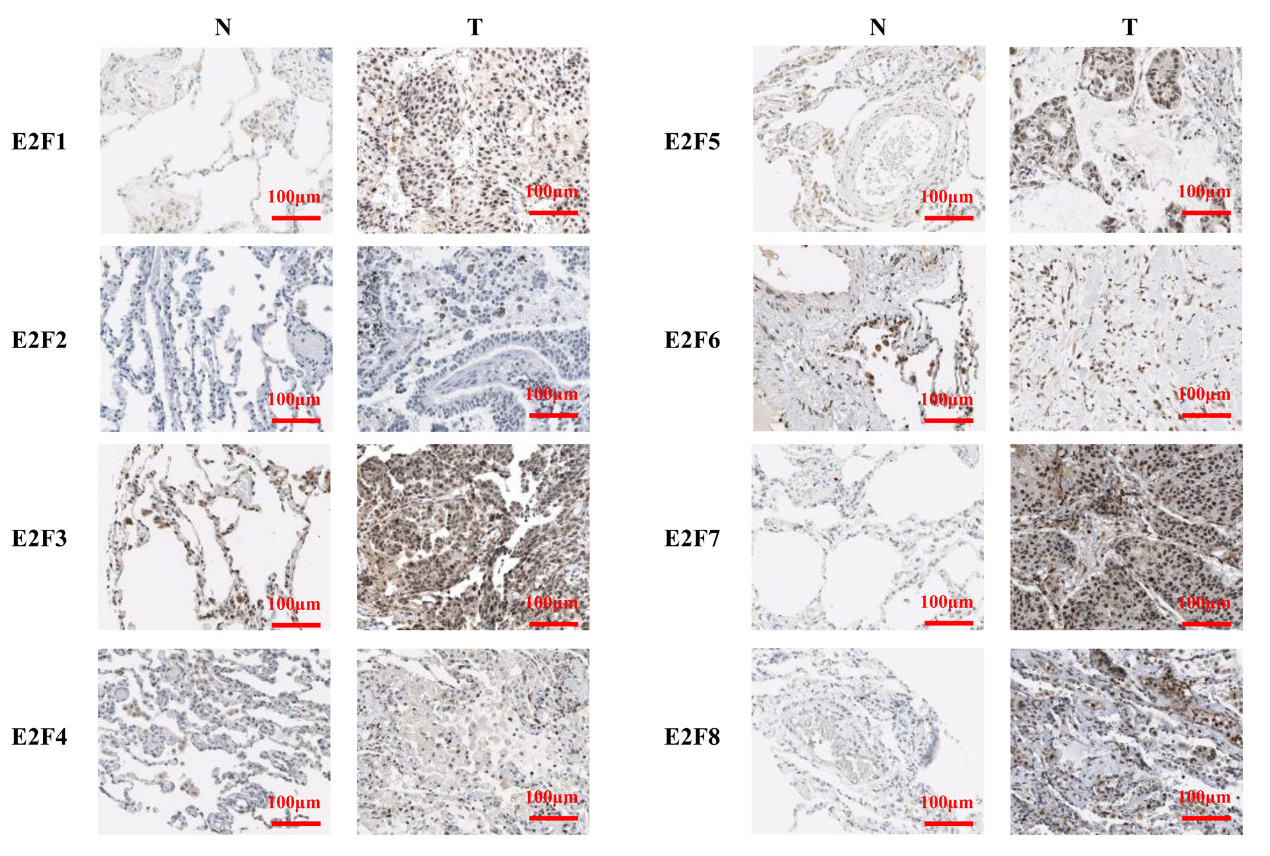
The expression of E2Fs in LC (IHC).

We performed IHC to test E2F proteins expression in LC tissues and their counterparts and to examine the expression of E2Fs in LC. We found that E2F1, E2F2, E2F7, and E2F8 proteins were more highly expressed in the LC tissues than in the normal lung tissues (Figure 4).

### Association of the increased mRNA expression of E2F1/2/4/5/7/8 and the decreased mRNA expression of E2F3/6 with the improved prognosis of patients with LC

**Figure 5:**
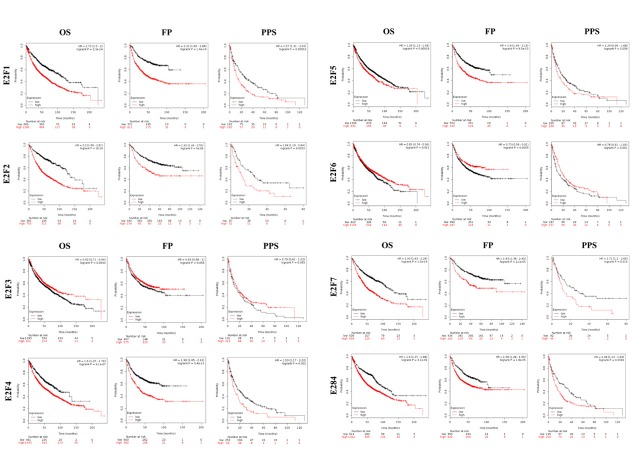
The prognostic value of mRNA level of E2F factors in LC patients (Kaplan-Meier plotter).

We further explored the critical efficiency of E2Fs in the survival of patients with NSCLC. Kaplan–Meier Plotter tools were used to analyze the correlation between the mRNA levels of E2Fs and the survival of patients with NSCLC in 2437 lung tumors by using publicly available datasets (2015 version) (http://kmplot.com/analysis/index.php?p=service&cancer=lung). The Kaplan–Meier curve and log-rank test analyses revealed that the increased E2F1/2/4/5/7/8 mRNA levels and the decreased E2F3/6 mRNA levels were significantly associated with the overall survival (OS), progression-free survival (FP), and post-progression survival (PPS) (*p* < 0.05) (Figure 5) of all of the patients with LC. The patients with LC with high mRNA levels of the E2F1/2/4/5/7/8 factors or low mRNA levels of E2F3/6 were predicted to have high OS, FP, and PPS.

### Predicted functions and pathways of the changes in E2F factors and their frequently altered neighbor genes in patients with LC

**Figure 6:**
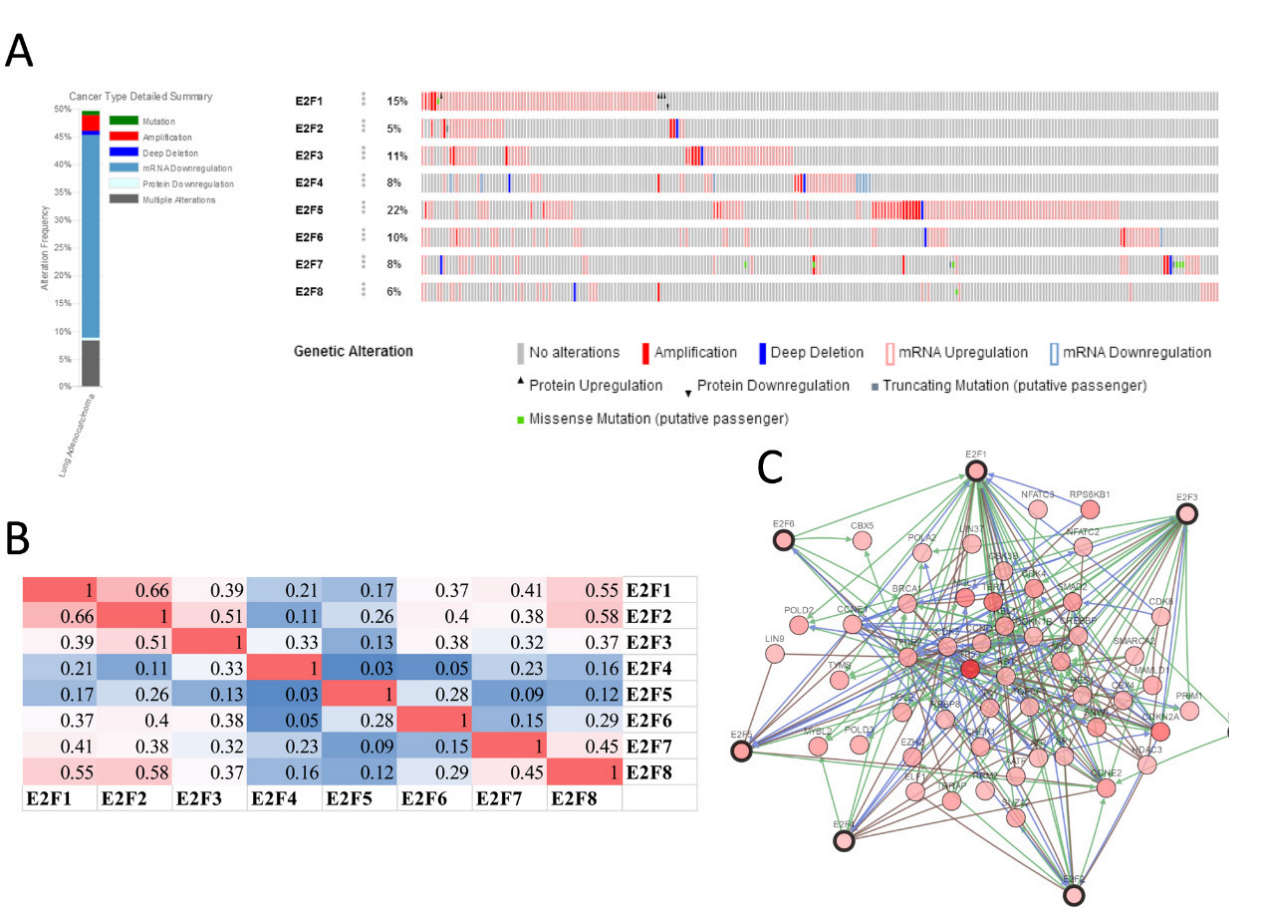
E2F genes expression and mutation analysis in lung adenocarcinoma (cBioPortal).

We analyzed the E2F alterations, correlations, and networks by using the cBioPortal online tool for lung adenocarcinoma (TCGA, Provisional). http://www.cbioportal.org/ index.do?session_id=5a37ba8e498eb8b3d56242fb. E2Fs were altered in 257 samples out of 522 patients with lung adenocarcinoma (49%). Two or more alterations were detected in almost half of the samples (128 samples) (Figure 6A). We also calculated the correlations of E2Fs with each other by analyzing their mRNA expression (RNA Seq V2 RSEM) via the cBioPortal online tool for lung adenocarcinoma (TCGA, Provisional), and Pearson’s correction was included. The results indicated significant and positive correlations in the following E2Fs: E2F1 with E2F2, E2F7, and E2F8; E2F2 with E2F1, E2F3, and E2F8; E2F3 with E2F2; E2F7 with E2F1 and E2F8; and E2F8 with E2F1, E2F2, and E2F7 (Figure 6B). We then constructed the network for E2Fs and the 50 most frequently altered neighbor genes. The results showed that the cell cycle-related genes, including CDK2, CDK4, CCNE1, CCNE2, CDKN1B, and CDKN2A, were closely associated with E2F alterations (Figure 6C).

**Figure 7:**
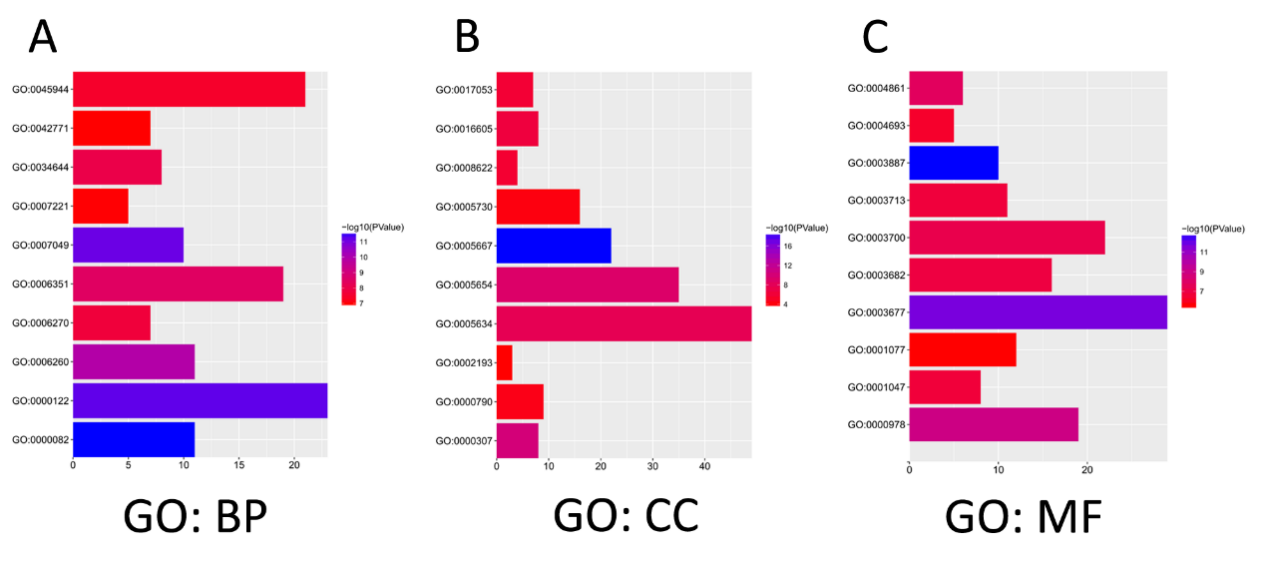
**The functions of E2Fs and genes significant associated with E2Fs alterations were predicted by analysis of Gene Ontology (GO) by DAVID (Database for Annotation, Visualization, and Integrated Discovery) tools (https://david.ncifcrf.gov/summary.jsp).** GO enrichment analysis predicted the functional roles of target host genes based on three aspects including biological processes (**A**), cellular components (**B**), and molecular functions (**C**).

The functions of E2Fs and the genes significantly associated with E2F alterations were predicted by analyzing Gene Ontology (GO) and Kyoto Encyclopedia of Genes and Genomes (KEGG) in the Database for Annotation, Visualization, and Integrated Discovery (DAVID) (https://david.ncifcrf.gov/summary.jsp). GO enrichment analysis predicted the functional roles of target host genes on the basis of three aspects, including biological processes, cellular components, and molecular functions. We found that GO:0007049 (cell cycle), GO:0000082 (G1/S transition of mitotic cell cycle), GO:0000122 (negative regulation of transcription from RNA polymerase II promoter), and GO:0045944 (positive regulation of transcription from RNA polymerase II promoter) were significantly regulated by the E2F alterations in lung adenocarcinoma (Figure 7A). GO:0005667 (TF complex), GO:0000307 (cyclin-dependent protein kinase holoenzyme complex), GO:0004693 (cyclin-dependent protein serine/threonine kinase activity), and GO:0004861 (cyclin-dependent protein serine/threonine kinase inhibitor activity) were also significantly controlled by these E2F alterations (Figures 7B and C). They are well-known genes associated with cell cycle.

**Figure 8:**
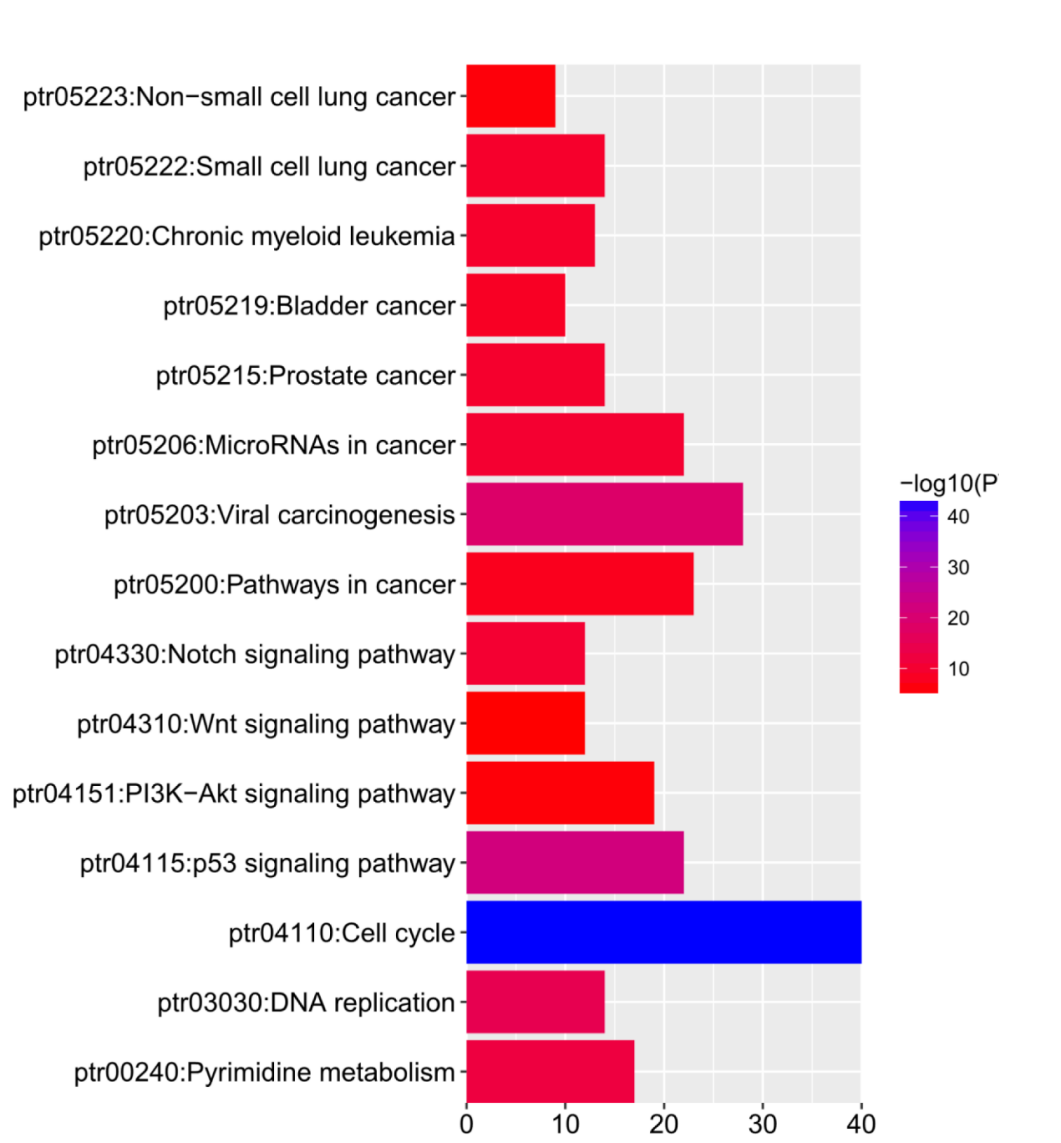
The functions of E2Fs and genes significant associated with E2Fs alterations were predicted by analysis of Kyoto Encyclopedia of Genes and Genomes (KEGG) by DAVID (Database for Annotation, Visualization, and Integrated Discovery) tools (https://david.ncifcrf.gov/summary.jsp).

**Figure 9:**
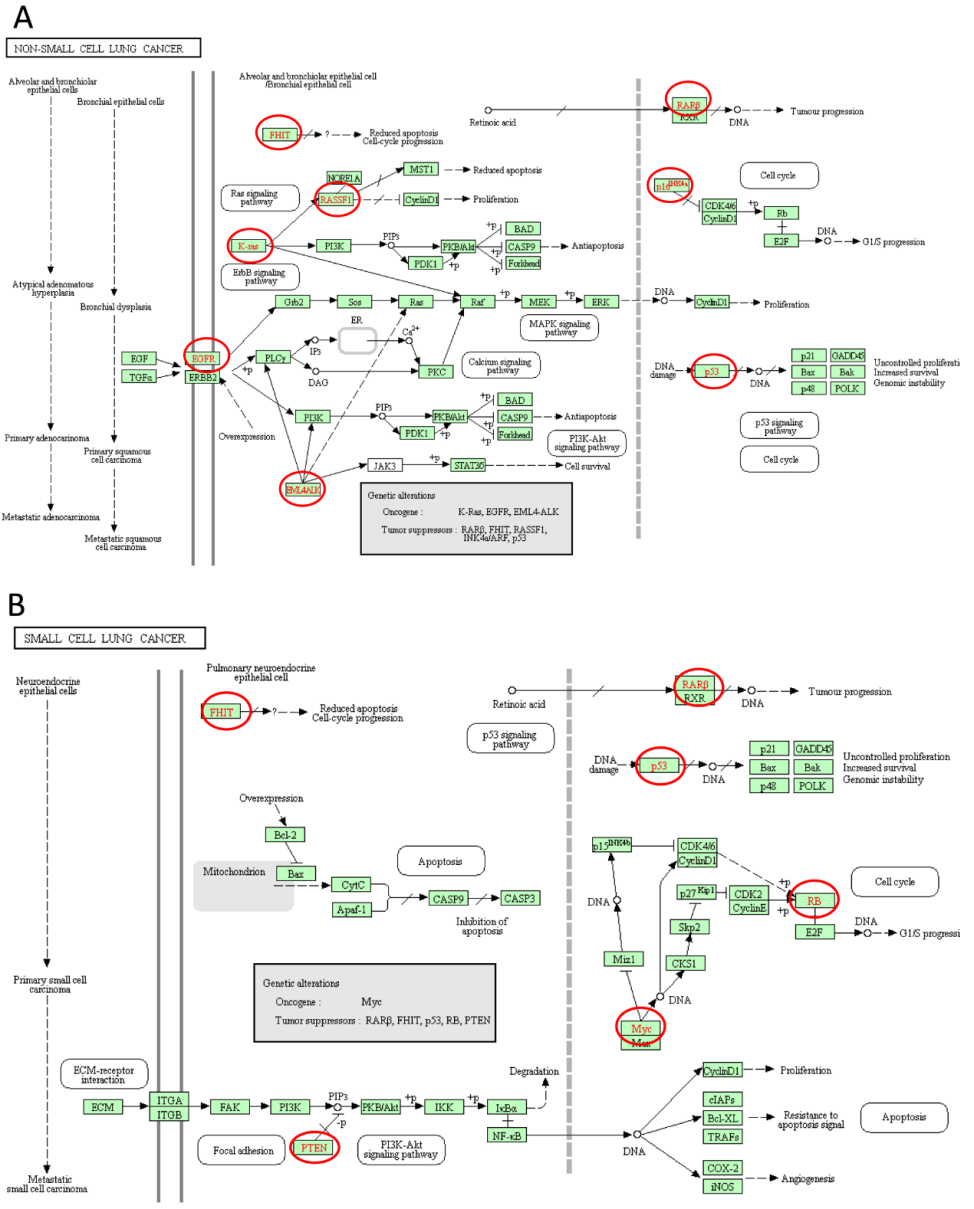
Non-small cell lung cancer and Small cell lung cancer signal pathways regulated by the E2Fs alteration in lung adenocarcinoma (cBioPortal).

KEGG analysis can define the pathways related to the functions of E2F alterations and the frequently altered neighbor genes. Fifteen pathways related to the functions of E2F alterations in lung adenocarcinoma were found through KEGG analysis (Figure 8). Among these pathways, ptr05223:NSCLC, ptr05222:SCLC, ptr05200:pathways in cancer, ptr04151:PI3K–Akt signaling pathway, ptr04310:Wnt signaling pathway, and ptr04330:Notch signaling pathway were involved in the tumorigenesis and pathogenesis of lung adenocarcinoma (Figures 9A and B).

## DISCUSSION

E2F factors dysregulation has been reported in many cancers [2,29–31]. Although the role of E2F activators in the tumorigenesis and prognosis of several cancers has been partially confirmed [7,18,19], further bioinformatics analysis of LC has yet to be performed. The present study is the first time to explore the mRNA expression and prognostic (OS, FP, and PPS) values of different E2F factors in LC. We hope that our findings will contribute to available knowledge, improve treatment designs, and enhance the accuracy of prognosis for patients with LC.

Among the E2Fs, E2F1 is the most studied in LC [32–38]. E2F1 overexpression contributes to the development of LC, and this role is enhanced by the deregulated pRb-p53-MDM2 circuitry [39]. E2F1 promotes EMT by regulating ZEB2 in SCLC [38]. In LC, some long noncoding RNAs (lncRNAs) and miRNAs perform their functions by regulating E2F1 [35,36]. E2F1 is also overexpressed in LC samples and involved in largazole-mediated cell cycle arrest at the G1 phase [34]. In our study, ONCOMINE datasets and TCGA datasets revealed that the expression of E2F1 was higher in human LC than in normal tissues. However, E2F1 expression was not correlated with the clinical characteristics of the patients with LC. Using the Kaplan–Meier Plotter, we determined the prognostic value of E2F1 in patients with LC. A high E2F1 expression was significantly associated with poor OS, FP, and PPS in all of the patients with LC followed up for 200 months.

E2F2 is a tumor activator in NSCLC and an independent indicator of OS in patients with NSCLC [18]. Feliciano et al. [40] reported that miR-99a represses EMT *in vivo* by inhibiting E2F2, prevents stemness features, and consequently decreases the number of cancer stem cells in LC. In our report, the expression of E2F2 in LC tissues was higher than that in normal tissues. We also demonstrated that E2F2 expression was significantly correlated with tumor stage in patients with LC. A high E2F2 expression was significantly correlated with poor OS, FP, and PPS in all of the patients with LC.

E2F3 overexpression is an oncogenic event during human LUAD and LUSC in patients with LC [41]. Trikha et al. [42] demonstrated that E2F3 participates as a key TF in tumor-associated macrophages and influences tumor microenvironment and lung cell metastasis. Interestingly, the lncRNA NEAT1 promotes NSCLC progression by acting as a ceRNA for miR-377-3p and then upregulating E2F3 [43]. Blood mRNA levels of E2F3 are significantly higher in patients with LC than in patients with benign lung diseases or healthy subjects, suggesting that the blood mRNA level of E2F3 is a sensitive diagnostic marker for LC [44]. In our report, we demonstrated that the expression of E2F3 in LC tissues was higher than that in normal tissues, but this expression was not correlated with tumor stage in patients with LC. But to our surprise, a low E2F3 expression was significantly correlated with poor OS, FP, and PPS in all of the patients with LC, which seemed inconsistent with the role of E2F3 as an oncogene.

E2F4, a member of the E2F family of TFs, is abundant in non-proliferating and differentiated cells, and TFs play important roles in the suppression of proliferation-associated genes [45]. E2F4 is a transcription repressor that inhibits cell proliferation and primarily mediates the anti-proliferative activity of capsaicin; the E2F4/p130 pathway has been implicated in the growth and progression of LC [46]. E2F4^−/−^ mice have defects in small airway epithelial cells, suggesting the role of this protein in lung development [47]. Bankovic et al. [48] studied genomic instability in patients with NSCLC through DNA fingerprinting and discovered that E2F4 is among the genes responsible for growth and metastasis of NSCLC. In our report, we demonstrated that the expression of E2F4 was lower in LC tissues than in normal tissues, but this expression was markedly correlated with tumor stage in patients with LC. Interestingly, a high E2F4 expression was significantly correlated with poor OS, FP, and PPS in all of the patients with LC. However, this finding seemed inconsistent with the role of E2F4 as a tumor suppressor.

E2F5 is found highly expressed in several tumors, such as glioblastoma [49], and prostate cancer [50]. But its expression and prognosis role in LC have not been reported. In this report, we demonstrated that the expression of E2F5 in LC tissues was higher than that in normal tissues, but this expression was markedly correlated with tumor stage in patients with LC. A higher E2F5 expression was significantly correlated with poor OS, FP, and PPS in all of the patients with LC.

E2F6 is highly expressed in NSCLC, and miR-424 can inhibit the proliferation and migration abilities of A549 cells by negatively regulating the expression of E2F6 [50]. E2F6 is also upregulated in NSCLC blood samples [51], indicating that E2F6 possesses an oncogenic feature in LC. However, the prognostic role of E2F6 in LC has yet to be investigated. In this report, we demonstrated that the expression of E2F6 in LC tissues was higher than that in normal tissues, but this expression was not correlated with tumor stage in patients with LC. A higher E2F6 expression was significantly correlated with poor OS, FP, and PPS in all of the patients with LC.

E2F7 and E2F8 function as transcriptional repressors [51]. They also likely serve as activators. E2F7/8 are shown to be activators of transcription; E2F8 binds to and activates the cyclin D1 promoter in a dominantly negative manner by blocking other E2Fs [52]. E2F7/8 directly bind to and stimulate the VEGFA promoter by cooperating with HIF1 [53]. ChIP-sequencing analysis revealed that E2F8 strongly binds to the promoter of UHRF1, and the identified sequence helps activate the promoter, showing that E2F8 may bind to and regulate its target genes, including UHRF1 [19]. E2F8 is also upregulated in LC, and si-E2F8 significantly represses tumor growth *in vivo* [55]. In the present study, E2F7 and E2F8 were significantly overexpressed in LC tissues, and their expression levels were markedly correlated with the tumor stage of the patients with LC. Interestingly, high E2F7/8 expression was significantly correlated with poor OS, FP, and PPS in all of the patients with LC, indicating the oncogenic role of TFs in LC.

In this study, we systemically analyzed the expression and prognostic value of E2Fs in LC and provided a thorough understanding of the heterogeneity and complexity of the molecular biological properties of LC. Our results indicated that the increased expression of E2F1/2/8 in LC tissues might play an important role in LC oncogenesis. High E2F1/2/7/8 expression could also serve as a molecular marker to identify high-risk subgroups of patients with LC. Our findings suggested that E2F1/2/5/8 were potential therapeutic targets for LC, and transcriptional E2F3/6/7 were potential prognostic markers for the improvement of LC survival and prognostic accuracy.

## MATERIALS AND METHODS

### Ethics statement

This study was approved by the Academic Committee of Wuhan University, and conducted according to the principles expressed in the Declaration of Helsinki. All the datasets were retrieved from the publishing literature, so it was confirmed that all written informed consent was obtained.

### ONCOMINE analysis

ONCOMINE gene expression array datasets (www.oncomine.org), an online cancer microarray database, was used to analyze the transcription levels of E2Fs in different cancers. The mRNA expressions of E2Fs in clinical cancer specimens were compared with that in normal controls, using a Students’ *t*-test to generate a *p* value. The cut-off of *p* value and fold change were defined as 0.01 and 2, respectively.

### GEPIA (Gene Expression Profiling Interactive Analysis) dataset

GEPIA is a newly developed interactive web server for analyzing the RNA sequencing expression data of 9,736 tumors and 8,587 normal samples from the TCGA and the GTEx projects, using a standard processing pipeline. GEPIA provides customizable functions such as tumor/normal differential expression analysis, profiling according to cancer types or pathological stages, patient survival analysis, similar gene detection, correlation analysis and dimensionality reduction analysis [54].

### The Kaplan-Meier plotter

The prognostic value of E2Fs mRNA expression was evaluated using an online database, Kaplan-Meier Plotter (www.kmplot.com) [55], which contained gene expression data and survival information of 2,437 clinical lung cancer patients (http://kmplot.com/analysis/index.php?p=service&cancer=lung). To analyze the overall survival (OS), progression-free survival (FP), and post progression survival (PPS) of patients with lung cancer, patient samples were split into two groups by median expression (high vs. low expression) and assessed by a Kaplan-Meier survival plot, with the hazard ratio (HR) with 95% confidence intervals (CI) and logrank *p* value. Only the JetSet best probe set of E2Fs were chosen to obtain Kaplan-Meier plots in which the Number-at-risk is indicated below the main plot.

### TCGA data and cBioPortal

The Cancer Genome Atlas had both sequencing and pathological data on 30 different cancers [56]. The lung adenocarcinoma (TCGA, Provisional) dataset including data from 522 cases with pathology reports was selected for further analyses of E2Fs using cBioPortal (http://www.cbioportal.org/index.do?session_id= 5a37ba8e498eb8b3d56242fb). The genomic profiles included mutations, putative copy-number alterations (CNA) from GISTIC, mRNA expression z-scores (RNA Seq V2 RSEM) and protein expression Z-scores (RPPA). Co-expression and network was calculated according to the cBioPortal's online instruction.

### Immunohistochemistry

3-μm tumor sections were incubated with commercial rabbit polyclonal antibodies against E2F1 (Santa Cruz), E2F2 (Santa Cruz), E2F3 (Santa Cruz), E2F4 (Santa Cruz), E2F5 (Santa Cruz), E2F6 (Santa Cruz), E2F7 (Santa Cruz), E2F8 (Santa Cruz) at 1/100 dilution overnight at 4°C. Then, the sections were conjugated with horseradish peroxidase (HRP) antibody (1:500 dilution; Santa Cruz Biotechnology, Santa Cruz, CA) at room temperature for 2 h, then covered by DAB (Vector Laboratories, Burlingame, CA), and slides were mounted with Vectashield mounting medium (Vector Laboratories). Subsequently, all fields were observed under light microscopy (Olympus 600 auto-biochemical analyzer, Tokyo, Japan). Control experiments without primary antibody demonstrated that the signals observed were specific.
